# CrossFit Motivates a 41-Year-Old Obese Man to Change His Lifestyle and Achieve Long-Term Health Improvements: A Case Report

**DOI:** 10.3390/jfmk8020058

**Published:** 2023-05-08

**Authors:** Tom Brandt, Timo Schinköthe, Annette Schmidt

**Affiliations:** 1 Institute of Sports Science, University of the Bundeswehr Munich, 85579 Neubiberg, Germany; 2 Comprehensive Cancer Center Munich CCCLMU, 81377 Munich, Germany

**Keywords:** high-intensity interval training, workplace health intervention, functional fitness, exercise, behavioural change and maintenance

## Abstract

The purpose of this case report was to demonstrate how CrossFit (CF) as a workplace health intervention (WHI) led to long-term lifestyle changes and health improvements in an inactive, sedentary individual. Therefore, we analysed the case of a 41-year-old obese man (BMI: 41.3 kg/m^2^) with elevated blood pressure and poor fitness. To evaluate the factors that facilitated his behavioural change, we collected quantitative and qualitative data (from 2015 to 2022) and analysed it based on the COM-B framework. Given the already great training opportunities at his workplace, we assumed that improvements in capability and motivation led to behavioural change and maintenance. Essential for this behavioural change was the fact that CF combined health-promoting training with intrinsically motivating aspects which are typical for classic sports such as challenge, a feeling of competence, and social interaction. In conjunction with rapid fitness improvements (capability), a positive feedback cycle between capability, motivation, and behaviour developed which enabled physical activity to become habitual. As a result, blood pressure was normalized, BMI (32.9 kg/m^2^) and resting heart rate decreased (−20 bpm), and mobility (FMS score: +89%), strength (+14 to 71%), and well-being (WHO-5 score: +12%) increased. In conclusion, CF should be considered an effective, efficient, and safe WHI with great potential for behavioural changes and maintenance.

## 1. Introduction

### 1.1. Changing Physical Activity Behaviour in the Workplace

Although there is clear evidence that an active lifestyle is beneficial for health and fitness, high proportions of adults do not meet the current physical activity (PA) guidelines [1,2]. An established strategy to improve physical activity and reduce sedentary behaviour is to implement workplace health interventions (WHI) [3]. Several recent studies reported positive outcomes for WHI [4,5,6]. However, previous research also indicated that the majority of employees did not participate in such programs at all and that positive effects were often not maintained after a program ended [7,8,9].

Guidance in the development of effective WHI can be provided by evidence-based behavioural change frameworks such as the COM-B framework [10]. According to this framework, it is the interaction between motivation, capability, and opportunity that ultimately determines the occurrence of target behaviour. On the other hand, behaviour also influences capability, opportunity, and motivation, which, in the long-term, creates feedback cycles. Motivation serves a key role in this system as it encompasses all brain processes that stimulate and control behaviour, including reflective and automatic processes. Nevertheless, the desired behaviour can only occur if the individual has sufficient physical and social opportunity (e.g., access to interventions, cultural milieu) as well as physical and psychological capability (e.g., health and fitness status, capability to engage in the required thought processes) [10]. Following this rationale, even excellent access to fitness classes and facilities, as well as an organization that encourages participation during working hours, may not be sufficient for employees with poor health and fitness to adopt an active lifestyle.

This was also the case with the participant of this report—a 41-year-old, obese Ph.D. student at the University of the Bundeswehr Munich (UniBw M). Despite diverse WHI (e.g., spinning, aqua fitness, yoga, circuit training, full body workout) and several sports facilities (e.g., gyms, swimming pool, outdoor calisthenics park, climbing hall) on campus that could be used free of charge during working hours he remained inactive until he encountered a WHI based on CrossFit^®^ (CrossFit, Inc., Washington DC, Columbia, USA) (CF). 

### 1.2. Potential of CrossFit^®^ as a Workplace Health Intervention

CF is a training program that incorporates constantly varied functional movements from different sports, such as weightlifting, gymnastics, and powerlifting, in high-intensity workouts [11]. Its rapid worldwide growth in popularity, high scalability, broad fitness adaptions, and clear health focus indicate that CF is an auspicious training concept for WHI to treat inactive, sedentary individuals with poor health and fitness [11,12]. While previous studies have already confirmed the positive physiological (e.g., body composition, cardiovascular/respiratory fitness, strength, flexibility, power, and balance) and psychological effects of CF, little is known about the behavioural change and maintenance qualities of CF-based interventions in the above-mentioned clientele [12,13,14].

In fact, some aspects of CF could impede participation due to their potentially detrimental effects on psychological variables [15]. For instance, if not monitored and adjusted adequately, training at high intensities and volumes could negatively affect participants’ moods [16,17]. Furthermore, it was found that the competitive practice of CF could translate into increased stress and worsened mood profiles [18]. On the other hand, recent studies have identified several motives for CF training participation, such as high levels of enjoyment, social support, challenge, and self-determination among individuals that already participated in CF [15,19,20,21]. However, when designing WHI, we must also ask what conditions, internal and external to the individual, need to be in place before the desired behaviour can occur at all.

Since the participant successfully changed his behaviour after he encountered CF and consequently gained health and fitness, we elaborate on his case in this report. Based on the COM-B framework, we aim to analyse which factors enabled his behavioural change and discuss whether CF was critical for this transition, providing guidance for future interventions.

## 2. Materials and Methods

### 2.1. Participants

As part of a clinical trial (trial registration at ClinicalTrials.gov, trial number: NCT05109286) investigating long-term effects of CrossFit (CF) in inactive adults with sedentary occupations [22], we recently evaluated a 41-year-old obese man (height: 1.86 m, weight: 142.9 kg; BMI: 41.3 kg/m^2^) with poor health and fitness. Until five years before participating in the study, he had an active lifestyle characterized by mountain sports such as climbing and mountain biking. After he began working as a full-time Ph.D. student at the University of the Bundeswehr Munich (UniBw M), he not only remained mostly sedentary during his working hours but also limited his leisure physical activities to weekends. The participant was aware of his unhealthy lifestyle and the need for behavioural change, given his severe obesity, hypertension, and low physical fitness. Although he had a positive attitude towards physical activity and knew about the workplace health interventions (WHI) at the UniBw M, he did not take advantage of the extensive training opportunities. On 6 December 2020, 3.5 months prior to study participation, he was forced to reduce his physical activity even further due to a bimalleolar ankle fracture with rupture of the syndesmosis (right leg).

### 2.2. CrossFit Intervention

The MedXFit-study provided two 60 min CF training sessions per week for 12 months. All training sessions were conducted at the military affiliate CrossFit Kokoro^®^ (CrossFit Kokoro-Military CrossFit Affiliation, Neubiberg, Germany). The training was led by experienced CF level 1 and 2 coaches who adjusted the program to the personal needs of the participants (maximum 10 participants per session) [22]. In the current case, the coaches paid special attention to the high body weight (142.9 kg) and just recently injured ankle of the participant. After study completion, he maintained CF training twice per week at CrossFit Kokoro^®^.

### 2.3. Data Collection

**Figure 1:**
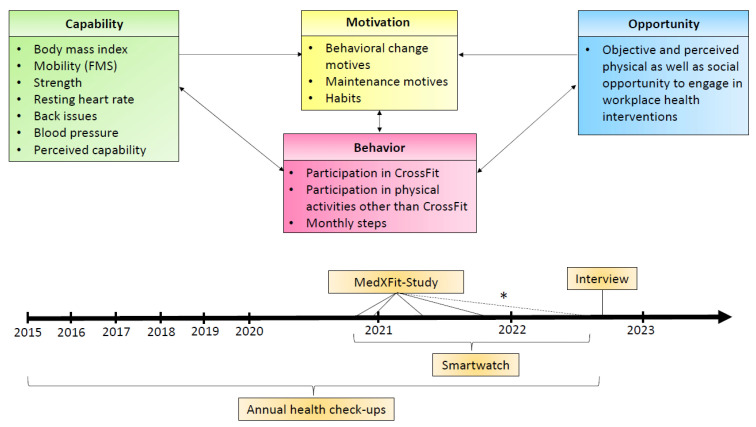
Chronical progression, important points, and measurement points in time. * Not part of the MedXFit-study but an identical test protocol.

We aim to present a comprehensive description of his transformation, covering all elements of the COM-B framework. Therefore, we collected data regarding his physical and social opportunities, physical and psychological capability, motivation, and behaviour [10]. This included data from the MedXFit-study, annual health check-ups from 2015 to 2022, an unstructured interview, and a smartwatch (Garmin Fenix 3) [22]. During the MedXFit-study, the participant was measured in October 2020, December 2020, April 2021, and October 2021. In July 2022, 9 months after the completion of the study, a follow-up with the same test protocol was carried out. The protocol included measuring anthropometrics (body weight and height with TANITA^®^ BC-545 (Tanita Corporation, Toyo, Japan) and SECA^®^ 213 scale (seca GmbH & Co. KG, Hamburg, Germany)), mobility (Functional Movement Screen), maximum isometric strength (Dr. WOLFF BackCheck^®^ 617 (Dr. WOLFF^®^ Sports & Prevention GmbH, Arnsberg, Germany)), well-being (WHO-5), and back-issues (questionnaire). The annual health check-ups (2015–2022) comprised a blood count and measurement of blood pressure (since 2017). The smartwatch (Garmin Fenix 3) tracked resting heart rate and steps per month. In addition to the bodyweight measurements during the MedXFit-study, his bodyweight was measured 2–4 times per month from July 2021–July 2022. Finally, a semi-structured interview was conducted with him on 23 September 2022. It included open-ended questions regarding his capability, opportunity, motivation, and behaviour before, during, and after encountering CF. The semi-structured interview guide can be found in Supplementary Materials Table S1. Figure 1 illustrates which data the different elements of the COM-B framework were mapped on and when we collected them.

The study was carried out according to the guidelines of the Declaration of Helsinki and was approved by the Institutional Ethics Committee of the UniBw M (6 April 2018). Informed consent was obtained from the participant before the study. The MedXFit-study is registered on ClinicalTrials.gov with the trial number NCT05109286.

## 3. Results

### 3.1. Capability

**Figure 2:**
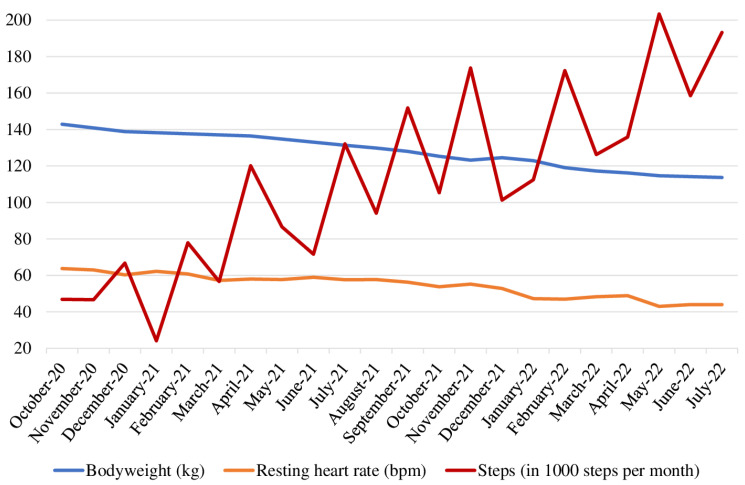
Bodyweight (kg), resting heart rate (bpm), and steps (in 1000 steps per month) from October 2020 to July 2022.

From October 2020 to July 2022, the participant’s body weight dropped from 142.9 (BMI: 41.3 kg/m^2^) to 113.7 kg (BMI: 32.9 kg/m^2^), and his resting heart rate decreased from 64 to 44 bpm (Figure 2).

**Figure 3:**
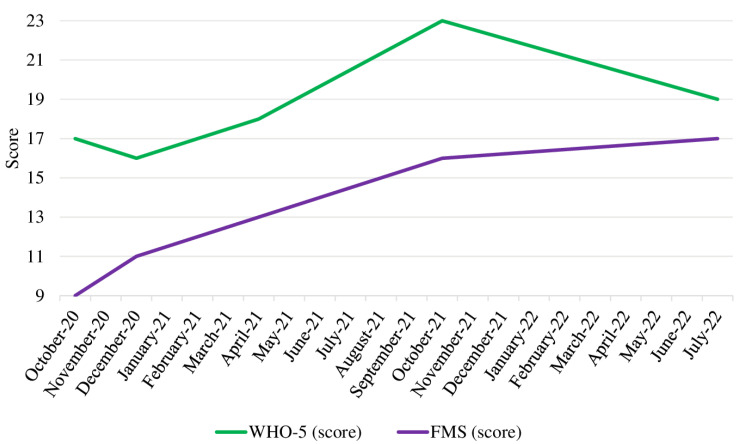
Functional Movement Screen and WHO-5 scores from October 2020 to July 2022. Note: FMS scores range from 0 to 21, WHO-5 scores range from 0 to 25. Abbreviations: FMS = Functional Movement Screen.

He also regained flexibility, coordination, and stability in his ankle, which, in turn, aided his performance in the FMS (17 points in June 2022) and during training (Figure 3).

**Figure 4:**
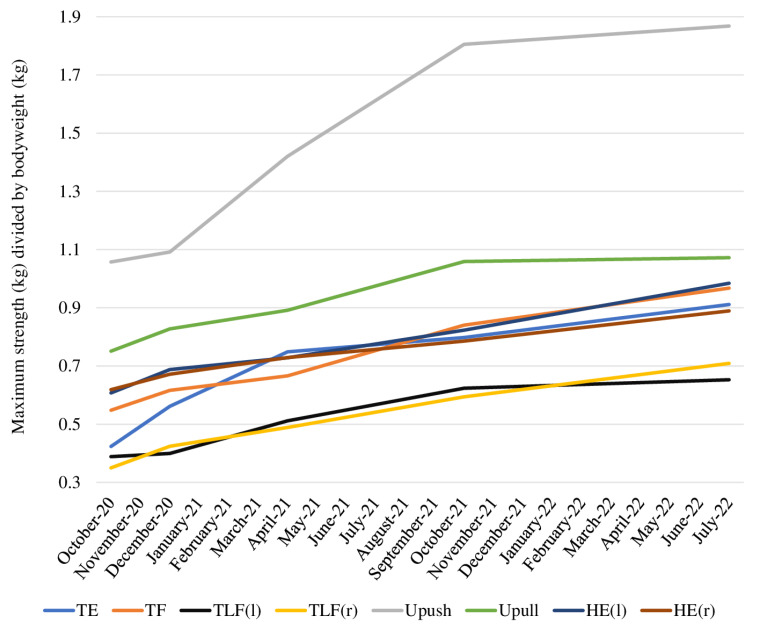
Relative strength calculated as maximum isometric strength (kg) divided by bodyweight (kg) from October 2020 to July 2022. Abbreviations: TE = trunk extension, TF = trunk flexion, TLF(l) = trunk lateral flexion left, TLF(r) = trunk lateral flexion right, UPush = upper body push, UPull = upper body pull, HE(l) = hip extension left, HE(r) = hip extension right.

Regarding the maximum isometric strength, we saw the greatest improvements during the first 12 months of the training program. From October 2021 until July 2022, the absolute numbers of the participant’s maximum isometric strength partly stalled. Nevertheless, he improved by 14–71% until July 2022, with the strongest improvement in the trunk extension (71%). Looking at his strength relative to body weight, he still showed improvements until the follow-up in July 2022 (Figure 4). 

An annual health check-up in 2021 revealed normal blood pressure (124/80 mmHg) for the first time since 2017. Based on the results from his annual health check-ups, he was advised to undergo a urine check by his primary physician due to elevated uric acid levels (2015: 7.5 mg/dl, 2016: 7.4 mg/dl) and erythrocytes in his urine (2017 and 2020: ery 2+). Additionally, the blood count in 2020 showed elevated levels of glutamate oxaloacetate transaminase (GOT) (86 U/l, reference range <35 U/l) and glutamate pyruvate transaminase (GPT) (56 U/l, reference range <45 U/l). Both values were within the reference range in 2021 (GOT: 30 U/l, reference range <50 U/l; GPT: 23 U/l, reference range: <50 U/l) and slightly elevated in 2022 (GOT: 58 U/l, reference range: <50 U/l; GPT: 53 U/l, reference range: <50 U/l). However, check-ups from 2021 and 2022 found no anomalies that would have required further investigations by his primary care physician. The results from the blood counts can be found in Supplementary Materials Table S2.

He has not reported any back issues since April 2021, and his well-being scores slightly improved until July 2022. Data from the MedXFit-study and his smartwatch are displayed in Table 1.

The participant stated that even before the study, he considered himself to be basically capable of engaging in workplace health interventions (WHI) at the University of the Bundeswehr Munich (UniBw M). Nevertheless, he mentioned a substantial improvement in his perceived physical capability, which were also evident in sports other than CrossFit (CF):

*“I feel stronger now. My endurance increased, and I can perform even complex movements like Muscle-Ups or Snatches now. I feel fitter while mountain biking and less fatigued after a tour.”*

In addition, the participant indicated that a lack of physical capability did not prevent him from participating in CF:

*“In CF, it is always possible to scale the training to your fitness level. Even when I am injured, I can train and adjust the training according to my needs or ask a coach what I should do.”*

### 3.2. Opportunity

The UniBw M offered its employees a wide range of sports activities (e.g., yoga, high-intensity training, aqua fitness, dancing, aerobic) as well as facilities (e.g., climbing hall, swimming pool, gym with cardio and weight rooms, calisthenics park) on campus free of charge. Per e-mail, information events, and flyers, the university actively encouraged employees to engage in WHI for 90 min per week during working hours. This was the case before, during, and after the completion of the MedXFit-study. The participant said it was important for him to train on campus in indoor facilities during his working hours. Even before the MedXFit-study, he was well informed about the training conditions at the UniBw M:

*“We received e-mails from the corporate health management on a regular basis during the past few years that promoted participation in some sort of physical activity. Furthermore, physical activity is fundamental at this university, as almost all students are active soldiers.”*

Among his colleagues and supervisors, the attitude toward sports was rated by him as neutral to positive. Regarding CF, he noted that there was a supportive atmosphere and close interaction between the coaches and participants:

*“Right from the start, there was a nice atmosphere during training. The trainers actively responded to the participants and adapted the training individually.”*

### 3.3. Motivation

The participant said that his weight gain accelerated after the ankle injury. This was a key motivator for him to start exercising again. CF was appealing to him because it combined health and performance aspects and could be conducted independently of weather conditions. Unlike other WHI on campus, he felt well informed about the training concept, although the MedXFit-study was his very first contact with CF. He never thought about quitting CF and stayed motivated due to the versatile and challenging training. Every time he learned a new exercise, it gave him extra motivation:

*“In CF, exercises and workout structure change constantly. It challenges you to learn new movements on a regular basis. After performing difficult movements for the first time, such as my first pullup or snatch, my motivation increased.”*

Furthermore, he mentioned the interaction with training partners and coaches, as well as the competitive nature of CF training, as important motivators. The vast number of classes per week and the need to sign up for them helped him to adhere to training. Although he stated that he enjoyed CF and is convinced of the training concept, he does not identify himself as a CrossFitter.

### 3.4. Behavior

Before the MedXFit-study, his physical activity was limited to cycling and rehabilitation exercises, both once per week. After study participation, he steadily increased his physical activity. While he completed 49 CF training sessions during the first 6 months of the study (1.9 training sessions per week) and occasionally mountain-biked (<1 training per week), he performed 67 CF training sessions (2.6 training sessions per week) during the second half of the MedXFit-study and mountain biked twice per week. After the MedXFit-study, he maintained CF training 2 days per week and mountain-biked once per week but added 2 Olympic weightlifting training sessions to his weekly schedule. Furthermore, he continuously increased his monthly step count throughout the observation period, although he remained mostly sedentary during his working hours (Figure 2).

## 4. Discussion

**Figure 5:**
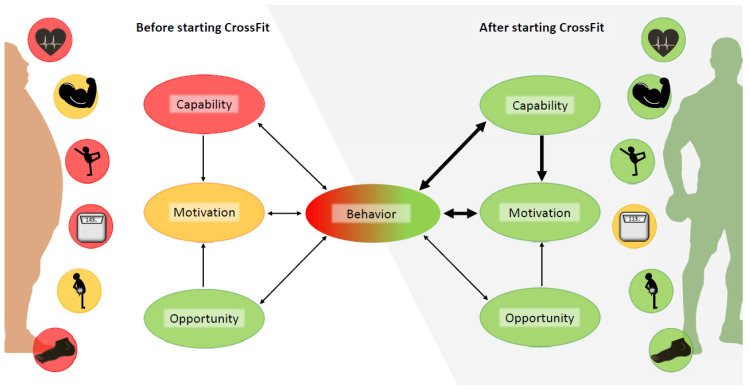
Changes within the components of the COM-B framework.

We have presented the case of a 41-year-old obese man living an inactive, sedentary life who successfully changed his behaviour after encountering CrossFit (CF) as part of a workplace health intervention (WHI). To identify the crucial internal and external conditions that facilitated his behavioural change and maintenance, we collected quantitative and qualitative data and analysed it based on the COM-B framework [10]. As target behaviour (physical activity, respectively CF) is an integral part of the COM-B framework but potentially affects opportunity, capability, and motivation after it occurs, we discuss factors for initial behavioural change separately from factors for behavioural maintenance. A schematic overview of our analysis results is provided in Figure 5.

### 4.1. Initial Behavioral Change

Predominantly sedentary jobs and insufficient leisure-time physical activity, as shown by the participant before he encountered CF, are common among university employees [23]. Looking at his baseline situation, it appears that he had great potential to be more physically active in the years before participating in the MedXFit-study [22]. In particular, his physical and social opportunities were at a high level. Major facilitators such as onsite exercise facilities, a great variety of group exercise classes or the possibility of exercising during working hours were already given at the University of the Bundeswehr Munich (UniBw M) [24,25]. Furthermore, he stated that the UniBw M provided a supportive social atmosphere towards physical activity. Because he remained inactive prior to the MedXFit-study, even though he valued the aspects mentioned above (on-site facilities, indoor training, exercise during work hours), we can conclude that opportunity did not initiate behavioural change but still served as an important prerequisite throughout the change process. Instead, we can assume that the interaction between capability and motivation was detrimental in his case.

Apart from well-developed maximum isometric strength, the participant showed distinct deficiencies regarding his physical capability. Of particular concern were his body weight (BMI: 41.3 kg/m^2^, obesity class III) and recent ankle injury, as these factors were found to be potential barriers to participation in physical activity [26,27]. His low physical capability was also evident in the Functional Movement Screen (FMS) score in October 2020 (9 out of 21 points). A score that low did not only quantify his inability to execute fundamental movement patterns but also indicated that he was prone to injury [28,29]. Elevated blood pressure and pain in the lower back were additional factors suggesting that he lacked physical capability prior to the MedXFit-study. Although he was aware of his low physical fitness, he considered himself to be basically capable of engaging in WHI. However, it was the ankle injury and subsequent rapid weight gain that led to dissatisfaction with his health status and finally prompted him to increase his physical activity. This implies that dissatisfaction might serve as a motivator and that physical capability is not as crucial for behavioural change in the first place as long as the individual feels physically capable. This finding is consistent with previous studies, which showed that experience or skills from past athletic activities limited the impact of decreased capability (e.g., after an injury) [27].

The fact that he chose CF among the numerous sports and fitness courses offered at the UniBw M could also be related to his athletic past. CF advertises itself as the sport of fitness and combines many factors that typically motivate athletes for sports participation [11,30]. While the participant considered the other courses as boring and mainly tailored to elderly individuals, he was attracted by the challenge, affiliation, and enjoyment CF seemed to offer. This suggests that health-focused interventions may benefit from incorporating elements of sports to initially change the behaviour of sedentary and inactive employees with a history of sports.

### 4.2. Behavioral Maintenance

Opportunity did not change substantially after October 2020. We can therefore assume that the interaction between behaviour, capability, and motivation primarily facilitated behavioural maintenance. While his occupation still required mostly sedentary behaviour, he steadily increased his physical activity outside of office time. With 1.9 CF classes per week during the first 6 months of the MedXFit-study, he met the guidelines for muscle and mobility-enhancing activities. In conjunction with occasional mountain biking, he also fulfilled the recommendation for aerobic exercise during most weeks [2]. The training was challenging for him but reasonably scaled to his physical capability. This provided him with effective training stimuli and led to fitness improvements that were in line with those found in previous studies [12,13,14]. Although he did not receive any other intervention (e.g., medication, diet), he continuously reduced his body weight and progressed in several other fitness measures, respectively, with physical capability (blood pressure, mobility, maximum isometric strength, well-being, resting heart rate).

The nature of CF—including scored workouts, competition, and the execution of complex movements—allowed him to experience and track his progress. This was important as he mentioned mastering new movements or achieving a high score during workouts to be exceptionally motivating. A steady increase in performance, the absence of overload symptoms and a gradual increase in physical activity outside of CF training further indicated that intensity and volume were in line with his capability. This hypothesis is supported by his perception regarding the scalability of CF in conjunction with the coaches’ assistance in case of performance deficits. We believe that this aided training enjoyment and that negative effects on mood associated with a high training volume or intensity were less likely to occur [16,17]. Additionally, the participant mentioned that he benefited from the competitive character of CF in terms of motivation rather than being exposed to additional stress [18]. It can, therefore, be assumed that his mood profited from CF training, which, in turn, may have had a positive effect on behavioural maintenance [31]. The high motivational potential of challenges, as well as a feeling of competence, have been found to be strong motivators among CF participants in previous research [21]. Because the participant saw steady progress, his long-term motivation profited from the frequent challenges during training. Nevertheless, progress-based motivation remains a double-edged sword, as the absence of progress might induce demotivation. Further sources of motivation which are independent of progress in physical capability are, therefore, mandatory for behavioural maintenance. For the participant, these especially included interactions with coaches and participants. In the MedXFit-study, training was conducted in small groups (<10 participants) under the supervision of a CF coach. This ensured social interaction between participants as well as high-quality coaching. While the former aided enjoyment and the commitment to show up for classes, the latter was crucial for his progress. Additionally, the participant valued the wide variety of equipment and exercises typically used in CF [11]. Overall, it appears that he experienced important motives for behavioural maintenance, such as satisfaction with behavioural outcomes, enjoyment in performing the behaviour, and the ability to cope with barriers [32]. In the context of the COM-B framework, it is, therefore, logical that, in conjunction with improved capability, he was able to steadily increase his physical activity, respectively, with target behaviour. This confirms the assumption that, in the long term, a feedback cycle between behaviour, motivation, and capability develops, and thus. participation in physical activity can become habitual. The formation of habits further increases the chance that the participant will continue to stay physically active in the future [32].

### 4.3. Limitations

These results are not generalizable because the case report included only one participant. In addition, it would have been advantageous to have qualitatively assessed his perception of motivation, capability, opportunity, and behaviour already before he changed his behaviour and throughout the training process.

## 5. Conclusions

The present report indicates that CF is an effective WHI for inactive, sedentary employees to improve their health in the long-term. It is essential for long-term behavioural changes that CF be combined health-promoting training with intrinsically motivating aspects that are usually found in classic sports are available. The main factors were motivation through challenges, feelings of competence, and social interaction. We hypothesize that especially former athletes, who could not be addressed by other health interventions or excellent training conditions, could benefit from CF-based interventions. A rapid increase in physical fitness (capability) proved to be beneficial as it supported the development of positive feedback cycles between capability, motivation, and behaviour up to the point where physical activity became habitual. Conversely, a lack of motivation due to stagnating performance represents a certain risk, but this could be compensated by the supportive social atmosphere and qualified coaching in CF. Efficiency and safety are additional arguments for considering CF in workplace settings, where heterogeneous groups need to be coached and training time competes with working time. We showed that even individuals with severely impaired fitness could safely attend group sessions and regain fitness with minimal time investment.

In conclusion, we suggest that health professionals should be encouraged to consider CF as an effective, efficient, and safe WHI with great potential for behavioural change and maintenance.

## Figures and Tables

**Table 1:** MedXFit-study and smartwatch data from October 2020 to July 2022. Abbreviations: BMI = body mass index, TE = trunk extension, TF = trunk flexion, TLF(l) = trunk lateral flexion left, TLF(r) = trunk lateral flexion right, UPush = upper body push, UPull = upper body pull, HE(l) = hip extension left, HE(r) = hip extension right, LBI = lower back issues.

Variable	October 2020	December 2020	April 2021	August 2021	October 2021	June 2022	July 2022
Bodyweight (kg)	142.9	138.9	136.5	129.8	125.3	114.2	113.7
BMI in kg/m^2^	41.3	40.2	39.5	37.5	36.2	33	32.9
Resting heart rate (bpm)	64	60	58	57	54	44	44
Steps/month	46,887	66,762	120,118	94,149	105,392	158,546	193,230
Well-being (WHO-5 score)	17	16	18		23		19
FMS (score)	9	11	13		16		17
TE (kg)	60.5	78	102		100		103.6
TF (kg)	78.3	85	90		105.3		110
TLF(l) (kg)	55.5	55	69		78.1		74.2
TLF(r) (kg)	50	58	66		74.4		80.6
UPush (kg)	151.1	151	193		226.2		212.4
UPull (kg)	107.3	114	121		132.7		121.9
HE(l) (kg)	86.8	95	99		103.1		111.9
HE(r) (kg)	88.4	93	99		98.4		101.1
LBI intensity (score)	3	2	0		0		0
LBI limitation (score)	3	3	0		0		0
LBI frequency days/week	1	3	0		0		0

## Data Availability

The data that support the findings of this study are available from the corresponding author upon reasonable request.

## Supplementary Materials

The following supporting information can be downloaded at: https://www.mdpi.com/article/10.3390/jfmk8020058/s1, Table S1: Semi-structured interview guide, Table S2: Results of blood counts from 2015 to 2022.
