# Supplementary material for: CrossFit Motivates a 41-Year-Old Obese Man to Change His Lifestyle and Achieve Long-Term Health Improvements: A Case Report
Source: J Funct Morphol Kinesiol. 2023 May 8;8(2):58. doi: 10.3390/jfmk8020058 (PMC10204440; doi:10.3390/jfmk8020058)
Supplement: Supplementary file 1 [file jfmk-08-00058-s001.zip › Table S1_Interview.pdf]

Table S1 Semi-structured interview guide.

Issue	Questions
Behavior	Describe the type and amount of physical activity you did before participating in the MedXFit study.<br><br>Before the MedXFit study, were you aware of the impact your physical activity has on your health?<br><br>Describe the type and amount of physical activity you did during and after the MedXFit-study.<br><br>What do you do when you can not train due to other events or injuries?
Capability	How would you rate your ability to exercise before participating in the MedXFit-study?<br><br>Did you think you were able to participate in other workplace health interventions offered at the University of the Bundeswehr in Munich before you participated in the MedXFit-study?<br><br>How did participating in the MedXFit-study impact your ability for engaging in physical activities?<br><br>In what situations did you perceive the changes in your ability to exercise?<br><br>How do you rate your health and fitness development from the beginning of the MedXFit-study until now?
Opportunity	Did you know about the sports facilities and courses at the University of the Bundeswehr in Munich before you participated in the MedXFit-study?<br><br>How do you rate the opportunity to engage in physical activities at the University of the Bundeswehr in Munich before, during, and after the start of the MedXFit-study?<br><br>How would you describe the attitude of the University of the Bundeswehr in Munich towards participation in physical activities before, during, and after the start of the MedXFit-study?<br><br>Describe the attitude of your social environment (colleagues, supervisors, ...) towards participation in physical activities during working hours before, during, and after you participated in the MedXFit-study.
Motivation	Why didn't you make use of the opportunities to be physically active at the University of the Bundeswehr in Munich before the MedXFit-study?<br><br>How did you become aware of the MedXFit-study?<br><br>What motivated you to participate in the MedXFit-study and try CrossFit?

	What motivated you for training during the MedXFit-study?<br><br>What role did the interaction with the other participants and coaches play regarding your motivation?<br><br>Did you ever think about quitting?<br><br>Were there any key moments that particularly motivated you?<br><br>What role did the CrossFit training concept play regarding your motivation?<br><br>Do you enjoy CrossFit and if so, has this been the case since the beginning of the MedXFit-study?<br><br>How do you rate the CrossFit training concept?<br><br>Do you see yourself as a CrossFitter?
