# Supplementary material for: CrossFit Motivates a 41-Year-Old Obese Man to Change His Lifestyle and Achieve Long-Term Health Improvements: A Case Report
Source: J Funct Morphol Kinesiol. 2023 May 8;8(2):58. doi: 10.3390/jfmk8020058 (PMC10204440; doi:10.3390/jfmk8020058)
Supplement: Supplementary file 1 [file jfmk-08-00058-s001.zip › Table S2_BAD.pdf]

Table S2 Results of blood counts from 2015 to 2022.

	Sep-15	Sep-16	Sep-17	Sep-18	Aug-19	Oct 20	Aug-21	Aug-22
Leukocytes G/l<br>(reference range)	7.4<br>(3.5-9.8)	7.2<br>(3.5-9.8)	6.3<br>(3.9-10.2)	6<br>(3.9-10.2)	6.9<br>(3.9-10.2)	6.5<br>(3.9-10.2)	9<br>(3.9-10.2)	6.4<br>(3.9-10.2)
Erythrocytes T/l<br>(reference range)	4.9<br>(4.5-5.9)	5<br>(4.5-5.9)	4.58<br>(4.3-5.75)	4.77<br>(4.3-5.75)	4.82<br>(4.3-5.75)	4.86<br>(4.3-5.75)	4.58<br>(4.3-5.75)	4.64<br>(4.3-5.75)
Hemoglobin g/dl<br>(reference range)	15.7<br>(13.5-17.5)	15.9<br>(13.5-17.5)	14.9<br>(13.5-17.2)	15.3<br>(13.5-17.2)	15.5<br>(13.5-17.2)	15.1<br>(13.5-17.2)	14.3<br>(13.5-17.2)	15.3<br>(13.5-17.2)
Hematocrit %<br>(reference range)	44.5<br>(40-53)	44.9<br>(40-53)	41.9<br>(39.5-50.5)	45.3<br>(39.5-50.5)	45.9<br>(39.5-50.5)	44.8<br>(39.5-50.5)	46.3<br>(39.5-50.5)	44.7<br>(39.5-50.5)
Mean corpuscular volume fl<br>(reference range)	90.3<br>(80-96)	90.3<br>(80-96)	91.5<br>(80-99)	94.9<br>(80-99)	95.2<br>(80-99)	92.2<br>(80-99)	101.1<br>(80-99)	96.3<br>(80-99)
Mean corpuscular hemoglobin pg<br>(reference range)	31.8<br>(28-33)	32<br>(28-33)	32.5<br>(27-33.5)	32.1<br>(27-33.5)	32.2<br>(27-33.5)	31.1<br>(27-33.5)	31.2<br>(27-33.5)	33<br>(27-33.5)
Mean corpuscular hemoglobin concentration g/dl<br>(reference range)	35.3<br>(32-36)	35.4<br>(32-36)	35.6<br>(31.5-36)	33.8<br>(31.5-36)	33.8<br>(31.5-36)	33.7<br>(31.5-36)	30.9<br>(31.5-36)	34.2<br>(31.5-36)
Thrombocytes G/l<br>(reference range)	214<br>(140-400)	237<br>(140-400)	241<br>(150-370)	195<br>(150-370)	216<br>(150-370)	234<br>(150-370)	241<br>(150-370)	204<br>(150-370)
Mean platelet volume fl<br>(reference range)			9.2<br>(5.9-9.9)	8.2<br>(5.9-9.9)	8.6<br>(7.4-11.7)	10.7<br>(7.4-11.7)	10.1<br>(7.4-11.7)	10.5<br>(7.4-11.7)
Red cell distribution %<br>(reference range)	13.2<br>(11.6-14.1)	12.9<br>(11.6-14.1)	13.9<br>(11.5-14.7)	13.2<br>(11.5-14.7)	13.6<br>(11-15)	13.5<br>(11-15)	14<br>(11-15)	13.2<br>(11-15)
Neutrophils %<br>(reference range)	49.3<br>(40-75)	49.7<br>(40-75)	57<br>(42-77)	56<br>(42-77)	51<br>(42-77)	54<br>(42-77)	69<br>(42-77)	60<br>(42-77)
Eosinophils %<br>(reference range)	3.6<br>(<5.5)	3<br>(<5.5)	5<br>(0-6)	3<br>(0-6)	4<br>(0-6)	3<br>(0-6)	2<br>(0-6)	2<br>(0-6)
Basophils %<br>(reference range)	0.5<br>(<1.5)	0.7<br>(<1.5)	1<br>(0-2)	1<br>(0-2)	1<br>(0-2)	1<br>(0-2)	1<br>(0-2)	1<br>(0-2)
Monocytes %<br>(reference range)	10.3<br>(4-11)	7.8<br>(4-11)	6<br>(2-10)	6<br>(2-10)	5<br>(2-10)	8<br>(2-10)	8<br>(2-10)	7<br>(2-10)
Lymphocytes %<br>(reference range)	36.3<br>(18-48)	38.8<br>(18-48)	31<br>(20-44)	34<br>(20-44)	39<br>(20-44)	33<br>(20-44)	21<br>(20-44)	31<br>(20-44)
Neutrophils (abs) G/l<br>(reference range)			3.59<br>(1.5-7.7)	3.36<br>(1.5-7.7)	3.52<br>(1.5-7.7)	3.51<br>(1.5-7.7)	6.21<br>(1.5-7.7)	3.84<br>(1.5-7.7)
Eosinophils (abs) G/l<br>(reference range)			0.32<br>(0-0.5)	0.18<br>(0-0.5)	0.28<br>(0-0.5)	0.2<br>(<0.5)	0.18<br>(<0.5)	0.13<br>(<0.5)
Basophils (abs) G/l<br>(reference range)			0.06<br>(0-0.2)	0.06<br>(0-0.2)	0.07<br>(0-0.2)	0.07<br>(<0.2)	0.09<br>(<0.2)	0.06<br>(<0.2)
Monocytes (abs) G/l<br>(reference range)			0.38<br>(0.1-0.9)	0.36<br>(0.1-0.9)	0.35<br>(0.1-0.9)	0.52<br>(0.1-0.9)	0.72<br>(0.1-0.9)	0.45<br>(0.1-0.9)
Lymphocytes (abs) G/l<br>(reference range)			1.95<br>(1.1-4.5)	2.04<br>(1.1-4.5)	2.69<br>(1.1-4.5)	2.15<br>(1.1-4.5)	1.89<br>(1.1-4.5)	1.98<br>(1.1-4.5)
GOT (AST) U/l<br>(reference range)	37<br>(<50)	49<br>(<50)	46<br>(<50)	31<br>(<50)	34<br>(<50)	86<br>(<35)	30<br>(<50)	58<br>(<50)
GPT (ALT) U/l	50	47	39	28	31	56	23	53

(reference range)	(<50)	(<50)	(<50)	(<50)	(<50)	(<45)	(<50)	(<50)
y-GT U/l<br>(reference range)	34<br>(<60)	32<br>(<60)	25<br>(<60)	27<br>(<60)	23<br>(<60)	36<br>(<60)	14<br>(<60)	19<br>(<60)
LDL-cholesterol mg/dl<br>(reference range)					149<br>(<160)			
Creatinine mg/dl<br>(reference range)	0.94<br>(0.7-1.3)	0.95<br>(0.7-1.3)	0.97<br>(0.7-1.2)	0.96<br>(0.7-1.2)	1.01<br>(0.7-1.2)	0.95<br>(0.72-1.25)	0.82<br>(0.72-1.25)	1.22<br>(0.72-1.25)
CKD-EPI ml/min/1.73m^2<br>(reference range)	104<br>(>90)	101.9<br>(>90)	99<br>(>60)	99<br>(>60)	93<br>(>60)	99<br>(>60)	109<br>(>60)	72<br>(>60)
Urea mg/dl<br>(reference range)	35<br>(10-50)	25<br>(10-50)	20<br>(19-44)	27<br>(19-44)	33<br>(19-44)	20<br>(19-44)	28<br>(19-44)	
Uric acid mg/dl<br>(reference range)	7.5<br>(3.6-7)	7.4<br>(3.6-7)						
Glucose (NaF)<br>(reference range)					93<br>(<100)			

Note: Results within the reference range are colored green. Values outside of the reference range are colored red.

Abbreviations: abs = absolute, GOT (AST) = glutamate oxaloacetate transaminase (aspartate aminotransferase), GPT (ALT) = glutamate pyruvate transaminase (alanine transaminase), y-GT = gamma-glutamyl transferase, LDL-cholesterol = low-density lipoprotein cholesterol, CKD-EPI = chronic kidney disease epidemiology collaboration
